# Identification and isolation of human testicular peritubular myoid cells and Leydig cells by a combination of ITGA9 and NGFR

**DOI:** 10.1186/s12958-025-01389-w

**Published:** 2025-05-31

**Authors:** Sha Han, Liangyu Zhao, Jiaqiang Luo, Chenkun Shi, Chencheng Yao, Zhiyong Ji, Junwei Xu, Shuai Xu, Ruhui Tian, Erlei Zhi, Yuhua Huang, Xin Liu, Yuchuan Zhou, Zhi Zhou, Zheng Li, Peng Li

**Affiliations:** 1 Department of Andrology, The Center for Men’s Health, Urologic Medical Center, Shanghai Key Laboratory of Reproductive Medicine, Shanghai General Hospital, Shanghai Jiao Tong University School of Medicine, Shanghai, 200080 China; 2 School of Life Science and Technology, Shanghai Tech University, Shanghai, 200120 China; 3 Department of Urology, The Fifth Affiliated Hospital of Sun Yat-sen University, Zhuhai, Guangdong Province 519000 China; 4 Guangdong Provincial Key Laboratory of Biomedical Imaging, The Fifth Affiliated Hospital, Sun Yat-sen University, Zhuhai, Guangdong Province 519000 China; 5 The International Peace Maternity and Child Health Hospital, Shanghai Jiao Tong University School of Medicine, Shanghai, 200030 China; 6 Department of Andrology, The First Affiliated Hospital of Hebei North University, Zhangjiakou, Heibei Province China

**Keywords:** ITGA9, NGFR, Testicular peritubular myoid cells, Adult Leydig cells

## Abstract

**Background:**

Testicular somatic cells play an important role in supporting spermatogenesis. Leydig cells (LCs) and peritubular myoid cells (PTMs) originate from a common progenitor population and show similar expression signatures in adulthood, making it difficult to distinguish and isolate the two in vitro.

**Methods:**

In this study, new surface markers for identifying adult LCs (ALCs) and PTMs were discovered by reanalyzing testicular single-cell dataset. Differential expressions of ITGA9 and NGFR were confirmed through immunofluorescence staining of human testes. A novel Fluorescence activated Cell Sorting (FACS) protocol is established for the isolation of ALCs and PTMs based on the two markers. Long-term culture of both cells were performed and their characteristics were characterized and explored.

**Results:**

ITGA9+ /NGFR + cells were positive for markers of PTMs (SMA, CNN1) and negative for markers of ALCs (HSD3B, STAR), and were able to form tubular and spheroid structures in vitro. In contrast, ITGA9-/NGFR + cells were positive for ALC markers and negative for PTM markers, and showed a capacity of testosterone production in vitro. Also, both cells were negative for Sertoli cell marker SOX9. When the two cells were cultured, they can expand for more than 15 passages.

**Conclusions:**

Our study established a novel and efficient method for identifying and isolating human ALCs and PTMs, which provides a great potential for researches of the two cell types in human.

**Supplementary Information:**

The online version contains supplementary material available at 10.1186/s12958-025-01389-w.

## Introduction

Spermatogenesis depends on a fully functional somatic microenvironment. The development of the supportive environment begins after birth and is completed after puberty [1]. In mice, a unique Nr5a1 + progenitor cell population at E10.5 was found to be capable of differentiating into fetal Leydig cells. These interstitial progenitor cells undergo gradual transcriptional changes that restrict their competence towards a steroidogenic fate and will differentiate into fetal LCs and PTMs [2]. In addition, a common progenitor for LCs and PTMs has been found in humans that expresses markers for both mature LCs and PTMs [3]. Adult LCs (ALCs) produce testosterone (T), which is critical for the maintenance of spermatogenesis in the testes and the male sexual characteristics of the body [4]. The decline in testosterone production with age indicates a direct impairment of Leydig cell function [5].There have been many attempts to isolate human stem Leydig cells (SLCs), and many surface protein markers of SLCs have been proposed, including NGFR [6], PDGFRα [7] and Endosialin [8]. However, the cellular localization of these markers remains controversial [9]. At the same time, the isolation of functional ALCs proves to be difficult and requires a large amount of testicular tissue [10]. Human testicular PTMs are smooth, muscle-like cells with contractile properties that surround the wall of the seminiferous tubules [11]. These cells have regulatory functions through the secretion of paracrine factors (GDNF, NGF) and physical functions through their contractile properties to support sperm transport [12]. Isolation and cultivation of human testicular PTMs has been reported [12–14]. However, the methods used cannot completely exclude possible contamination by vascular smooth muscle cells and other somatic cells, so that it usually takes more than 4 weeks until the PTMs are well established in vitro [13]. The isolation and cultivation of human somatic testicular cells, such as LCs, PTMs and SCs, offers the unique opportunity to better study their function in vitro. However, it is still a challenge to isolate and culture the cells with good purity and function. Single-cell RNA sequencing (scRNA-seq) can effectively delineate cell types and identify cell markers [15]. We reanalyzed the previously published single-cell transcriptome data from human testis [1] and found that NGFR and ITGA9 have the potential to be used as surface markers of ALCs and PTMs. Here, we report our work on the expression of NGFR and ITGA9 in adult human testicular cells and the subsequent evaluation of the feasibility of applying the two markers to isolate human adult LCs and PTMs. Finally, we also characterized and evaluated the functions of the two cells after their long-term cultures in vitro.

## Materials and methods

### Donor testicular tissue

Human testicular tissues were retrieved from three obstructive azoospermia(OA) patients who underwent micro-dissection testicular sperm extraction with normal spermatogenesis, as determined by histology after written informed consent at the Shanghai General Hospital. The one specimens approximately 5 mm×5 mm×5 mm were immediately transported to the laboratory on ice and washed to remove any residual blood.

### Single-cell RNA sequencing (scRNA-seq) analysis

The detailed analysis was described previously [1]. In brief, single testicular cells were isolated from human testes by enzyme digestion, which were loaded on a Chromium Single Cell Controller instrument (10×Genomics) to generate single-cell gel beads in emulsions. The scRNA-seq libraries were prepared using the Chromium Single Cell 3’ Library & Gel Bead Kit. After mapping, sample quality control, and integration, 16 clusters of all testicular cells were identified. Unique cluster-specific marker genes were identified using the Seurat FindAllMarkers tool, which is based on the normalized UMI count.

### Immunofluorescence

For immunofluorescence staining, cells were fixed with 4% PFA for 30 min, washed three times with cold PBS (Gibco), and permeabilized with 0.4% Triton X-100 (Sigma) for 5 min. After extensive wash with PBS, the cells were blocked in 5% bovine serum albumin (BSA, Sigma) for an hour at room temperature. The cells were then incubated with primary antibodies overnight at 4 ℃ at the following dilutions: goat polyclonal anti-ITGA9 (dilution 1:100, R&D AF3827 [16], rabbit polyclonal anti-NGFR (dilution 1:500, ABCAM AB8874 [17], rabbit polyclonal anti-CYP11A (dilution 1:200, Proteintech [18], rabbit polyclonal anti-CYP17A (dilution 1:200, Novus NBP2-13892 [18], rabbit polyclonal anti-SMA (dilution 1:200, Servicebio GB111364 [19], rabbit polyclonal anti-CNN1 (dilution 1:200, Servicebio GB11722), mouse monoclonal anti-Nestin(NES) (dilution 1:200, Santa Cruz [20], mouse monoclonal anti-StAR (dilution 1:200, Santa Cruz sc-166821 [21], mouse polyclonal anti-HSD3B (dilution 1:200, Santa Cruz sc-100466 [22], rabbit polyclonal anti-SOX9 (dilution 1:200, Millipore AB5535 [18]. Antigen detection was conducted using the appropriate combination of Alexa Fluor 488 and 594 secondary antibodies for 1 h at room temperature in the dark. Hoechst was used to label the nuclei. Images were captured with an OLYMPUS confocal microscope.

### Immunostaining of testicular tissues

The immunofluorescence (IF) staining was performed on 5 μm formalin-fixed paraffin embedded (FFPE) sections from portions of the collected testicular and seminoma samples following deparaffinisation, rehydratation and heat-mediated antigen retrieval in 10 mM sodium citrate buffer solution (pH = 6). After treatment with 5% BSA for 1 h at room temperature, individual sections were incubated overnight at 4 ℃ with a mix of diluted antibodies. Antigen detection was conducted using the appropriate combination of Alexa Fluor 488 and 594 secondary antibodies (dilution: 1:500, Life Technologies/ThermoFisher Scientific) for 1 h at room temperature in the dark. As a negative control, the samples went through the same treatment process as the experimental group, but the primary antibody was omitted from the incubation step. Hoechst was used to label the nuclei. Images were captured with an OLYMPUS confocal microscope.

### Fluorescence activated cell sorting and cell culture

Human primary cells were isolated from testicular tissue measuring approximately 5 mm×5 mm×5 mm of clinical donors. In detail, the testes were mechanically cut and enzymatically disassociated with 2 mg/ml type IV collagenase (Gibco, Grand Island, NY, USA) in Dulbecco’s modified Eagle medium/nutrient mixture F-12 (DMEM/F12; 1:1, Gibco) at 37 °C for 15 min with slow shaking (100 cycles/min). The suspension was filtered through a 40 μm filter and centrifuged at 300 g for 3 min, and the supernatant was removed. The precipitant was resuspended in DMEM/F-12 with 10% fetal bovine serum (FBS, Gibco) and LC and PTM were attached to 6 cm culture dishes. After culture for 7 days. cells in 6 cm culture dish with 85-90% cell fusion were enzymatically disassociated with 0.25% Trypsin (Gibco) and centrifuged at 300 g for 3 min and the supernatant was removed. The cell pellets were rinsed two times with Ca^2+^-/Mg^2+^-free phosphate-buffered saline and then incubated with anti-NGFR and anti-ITGA9 antibody for 60 min and centrifuged at 300 g for 3 min. Then the cell pellets were resuspended by Ca^2+^-/Mg^2+^-free phosphate-buffered saline and was incubated anti-Rabbit and anti-Goat antibody in the dark for 60 min. The NGFR+/ITGA9- and NGFR-/ITGA9 + cells were enriched by flow cytometry (Influx Cell Sorter, BD Biosciences) followed by culture in expansion medium composed of DMEM/F12 with 10% FBS, 1% GlutaMAX (Gibco). The cells were cultured at 37 ℃  with 5% CO_2_, and the medium was changed every 2 days.

### RNA extraction, RT-PCR, and real-time qPCR

Total RNA was extracted from cultured cells or tissues using TRIzol (Takara, Kusatsu, Japan), and the quality and concentrations of total RNA were measured by NanoDrop (Thermo Fisher Scientific, USA). The ratio of A260/A280 of total RNA was set as 1.9 ~ 2.0 to ensure quality.

Reverse transcription (RT) of total RNA was conducted using the First Strand cDNA Synthesis Kit (Thermo Fisher Scientific, USA), and PCR of the cDNA was carried out according to the protocol as described previously. The primer sequences of chosen genes were designed and listed in Table S1. The PCR started at 94 ℃ for 2 min and was performed in terms of the following conditions: denaturation at 94 ℃ for 30 s, annealing at 55 ℃~60 ℃ for 45 s, and elongation at 72 ℃ for 45 s, for 35 cycles. The samples were incubated for an additional 5 min at 72 ℃. PCR with PBS but without cDNA served as a negative control. PCR products were separated by electrophoresis on 2% agarose gel and visualized with ethidium bromide. Images were recorded and band intensity was analyzed using chemiluminescence (Chemi-Doc XRS; Bio-Rad).

### ELISA

The hormone levels in the cell culture supernatants were measured using a commercially available ELISA kit (R&D) according to the manufacturer’s instructions. The absorbance at 450 nm was measured using an ELISA microtiter plate reader (Tecan, Switzerland). The hormone concentrations were evaluated according to a standard curve constructed by plotting the absorbance of each reference standard against its corresponding concentration. The coefficient of variation of this ELISA kit is 2.9–4% for intra-assay precision and 5.6–6.8% for inter-assay precision.

### Tube formation assays

Matrigel (BD Biosciences)-coated µ-Slide angiogenesis (ibidi GmbH) was used to observed tube formation of primary NGFR+/ITGA9- and NGFR+/ITGA9 + cells. Then 1 × 10^4^ primary NGFR+/ITGA9- and NGFR+/ITGA9 + cells in 50 µl of cultured medium were seeded. At 2 h after seeding, images were collected using light microscopy. The total tube length was measured and analyzed with Image-Pro-Plus 6.0.

### Generation of 3D cell spheroids in microwell culture

Generation of 3D cell spheroids in microwell culture was operated as described previously [23]. AggreWell 400 plates (STEMCELL Technologies Inc, Vancouver, Canada, cat# 34450) were prepared according to the manufacturer’s instructions, washed once with 0.5 mL PBS, and 0.5 mL of culture medium (Dulbecco Modified Eagle Medium F/12 supplemented with 10% FBS, 1% Penicillin Streptomycin) was placed in the wells. Each plate was centrifuged at 2000 g for 2 min to remove trapped air, 6 × 10^5^ (500 cell organoids) cells suspended in 0.5 mL medium were placed into each well, and the plates were centrifuged at 500×g for 5 min. The forming organoids were maintained (1 mL medium/well) in microwell plates for 2 days at 37 ℃ in 5% CO_2_ in air with half medium change every day. The number of cell spheroids were counted and cell spheroids index was calculated by dividing the number of cell spheroids by the total (400).

### Tubule crawling method

To isolate PTM with traditional tubule crawling method as described previously [12, 13]. Testicular tissue covered with medium was dissected using tweezers under sterile conditions into 1- to 2-mm^3^ pieces. The tissue was then placed onto the surface of a plastic cell culture dish. The explants were incubated for 1–2 h under humidified conditions (37 ℃, 5% CO_2_), checked for adherence, and subsequently cultured in medium composed as described above. When the cells covered an area of approximately 1 cm^2^, the remnant explant was carefully removed, and cells were allowed to grow before they were trypsinized and subcultured.

### Statistical analyses

All data were analyzed using GraphPad Prism 6.0 (GraphPad Software) and results were presented as the mean ± standard deviation (SD). The Student’s t-test was used to analyze the differences between two groups. Analysis of variance (ANOVA) and a two-tailed t test were used to analyze the differences between multiple groups. All the experiments were performed independently at least three times. All statistical tests were two-tailed and P value < 0.05 was considered a statistically significant difference.

## Results

### Expression profile of ITGA9 and NGFR in human testis

To identify potential specific cell surface markers of human PTMs and ALCs. We reanalyzed our single-cell transcriptome data from OA testis tissue. In the whole cell population of 10 healthy volunteers, a total of nine cell clusters were identified based on the expression of known cell type-specific markers (Fig. S1A). These nine clusters included germ cells, endothelial cells, macrophages, vascular smooth muscle cells (VSMs), PTMs, Sertoli cells and ALCs. A total of 1,101 differentially expressed genes (DEGs) were identified with a fold change of the log2 transformed UMI > 1 of each cluster. The results of Gene Ontology (GO) analysis of these DEGs were consistent with our understanding of the biological processes of these cell types. NGFR is expressed by both PTMs and ALCs, while ITGA9 is specifically expressed by PTMs (Fig. S1B, C).

**Fig. 1:**
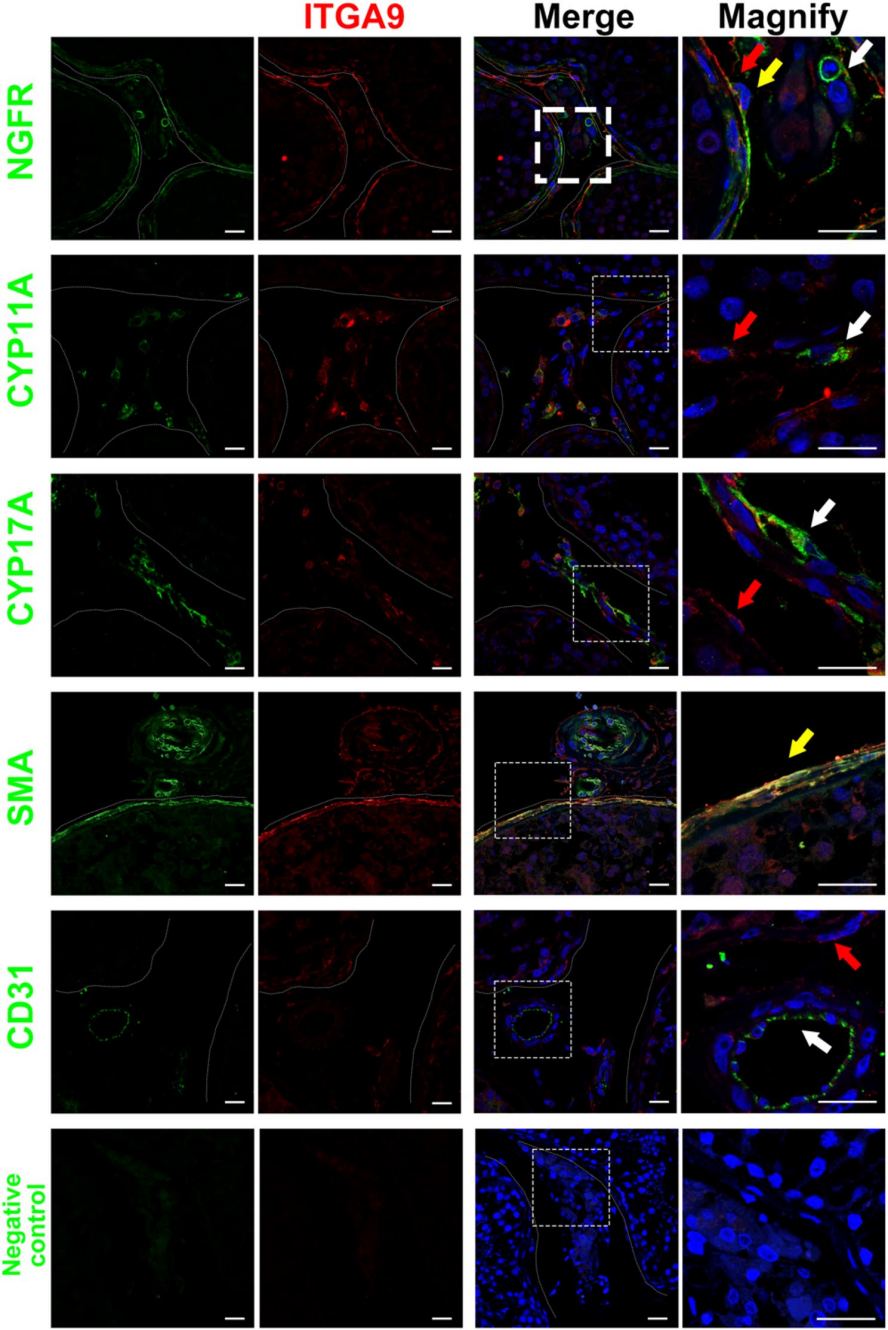
Immunofluorescence co-staining of ITGA9 (red arrow) with NGFR, CYP11A, CYP17A, SMA and CD31 (white arrow) in human testicular paraffin sections. Cells that are co-staining positive are indicated by yellow arrows. The scale bar represents 20 μm

We then examined the expression profile of ITGA9 and NGFR in adult human testis to determine whether ITGA9 and NGFR could serve as putative markers for human ALCs and PTMs. We found that both NGFR and ITGA9 were expressed exclusively in the interstitial regions of the testis. However, NGFR was expressed on both PTMs and ALCs, whereas ITGA9 was expressed exclusively on PTMs (Fig. S1D). Immunofluorescence co-staining showed that ITGA9 did not co-stain with the markers of ALCs (CYP11A, CYP17A) and vascular endothelial cells (VECs) (CD31) (Fig. 1). This provides us with a rational way to sort PTMs and ALCs based on these markers.

### Isolation and identification of ITGA9 or NGFR positive cells in vitro

**Fig. 2:**
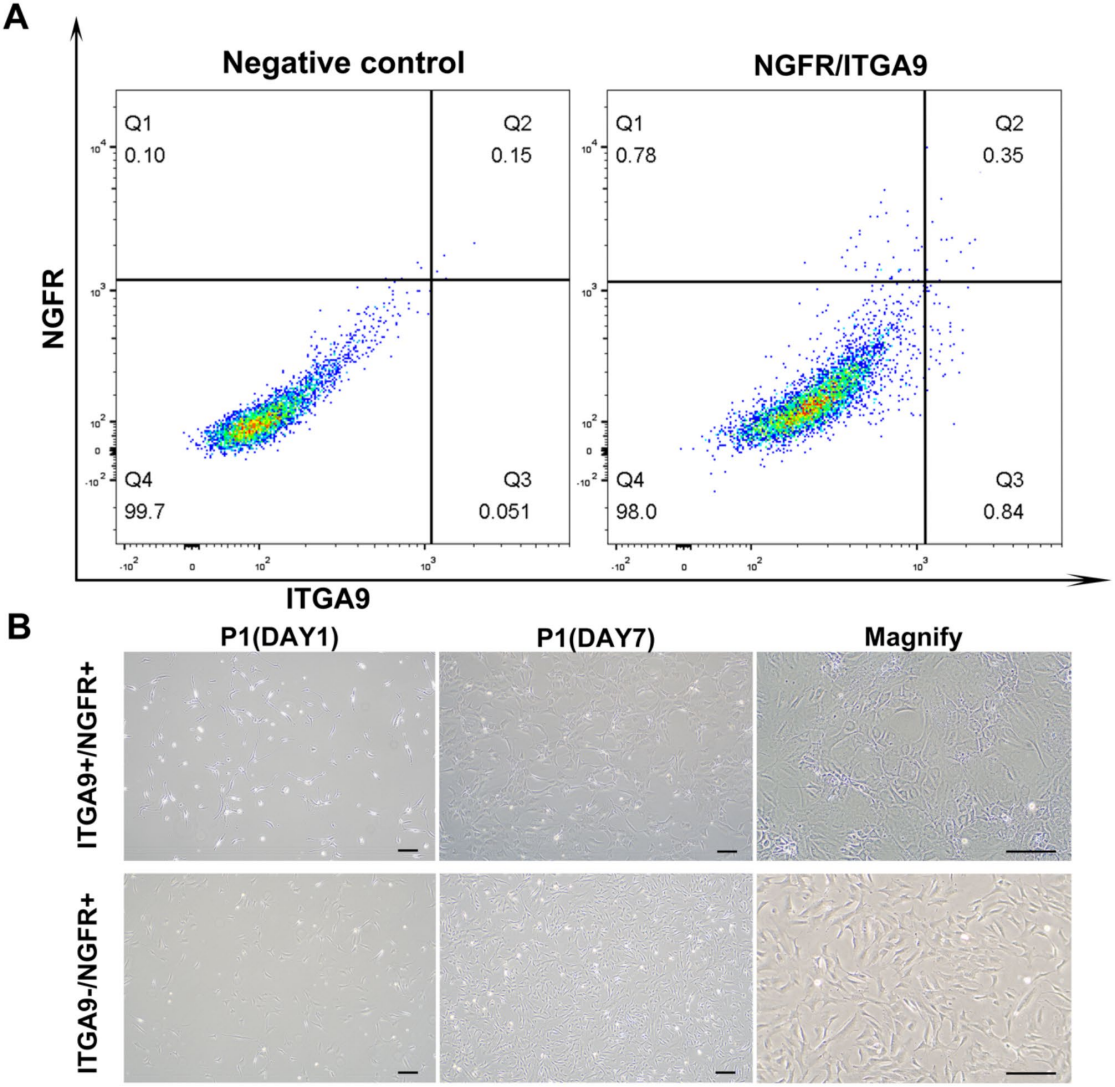
Isolation and proliferation of ITGA9 and NGFR cells in vitro. (**A**) ITGA9+/NGFR + and ITGA9-/NGFR + cells were isolated by FACS from adult human testes. (**B**) ITGA9+/NGFR + and ITGA9-/NGFR + cells were cultured and proliferation in vitro*.* Different cell morphologies can be seen. The scale bar represents 20 μm

To further investigate the cell-specific expression of ITGA9 and NGFR, human testicular cells were isolated by fluorescence-activated cell sorting (FACS). ITGA9+/NGFR + cells accounted for 0.2% of the total testicular cell population and ITGA9-/NGFR + cells accounted for 0.68% (Fig. 2A). These freshly isolated cells were then cultured separately on plastic dishes in expansion medium at a density of 2 × 10^5^/ml. Both cell types can continue to proliferate and the degree of confluence can reach 70–80% in about 7 days (Fig. 2B). It was found that ITGA9+/NGFR + cells grew in a ring pattern after a high degree of confluence (Fig. 2B).

### Long-term culture of ITGA9+/NGFR + cells in vitro

**Fig. 3:**
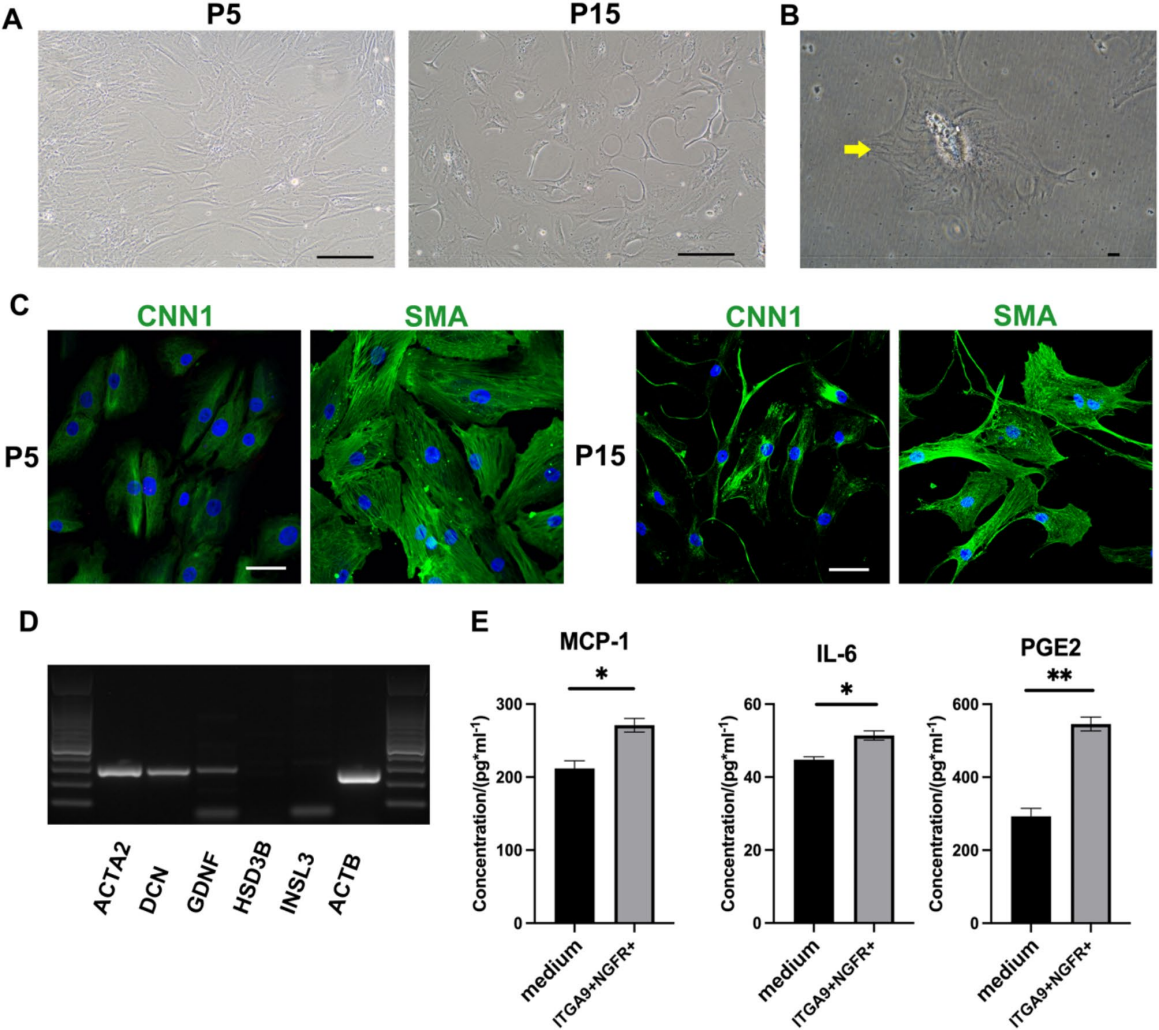
Identification of ITGA9+/NGFR + cells in vitro*.* (**A**) There was no obvious change in cell morphology after 15 passages under light microscope. (**B**) Myoid fibers texture can be found in ITGA9+/NGFR + cells. (**C**) ITGA9+/NGFR + cells expressed SMA, CNN1. (**D**) RT-PCR show that ITGA9+/NGFR + cells express PTM-specific genes ACTA2, DCN and GDNF, but do not express ALC-specific genes (HSD3B and INSL3). (**E**) The concentration of MCP-1, IL-6 and PGE2 in ITGA9+/NGFR + cells culture medium increased significantly

To clarify the purity and characterize the functions of ITGA9+/NGFR + cells in vitro, we cultured the cells for a prolonged period of time. ITGA9+/NGFR + cells could be passaged up to the 15th passage (Fig. 3A). The texture of the myoid fibers can also be seen under a microscope with high magnification (Fig. 3B). Subsequently, the sorted cells were characterized by immunostaining at the P5 stage when they had proliferated to 50–60% on plastic dishes. The ITGA9+/NGFR + cells expressed PTM markers (SMA and CNN1) (Fig. 3C), but were negative for markers of LCs (NES, INSL3, STAR) (Fig. S2A). Immunofluorescence staining of the cells after 15 passages also showed that the cells were still able to express the PTM markers CNN1 and SMA (Fig. 3C). At the same time, compared to the previous PTM isolation method [12, 13], the current method is more efficient. For example, with the traditional tubule crawling method, it took more than two weeks for the cells to reach 70–80% confluence, and the culture contained at least two cell types in the 1st and 5th passage (Fig. S3A). In addition, immunofluorescence staining showed that the PTMs extracted by the traditional method were contaminated with NES-positive cells (Fig. S3B). The purity of PTMs was significantly lower than the new FACS method used in this study (*P* < 0.01) (Fig. S3C).

**Fig. 4:**
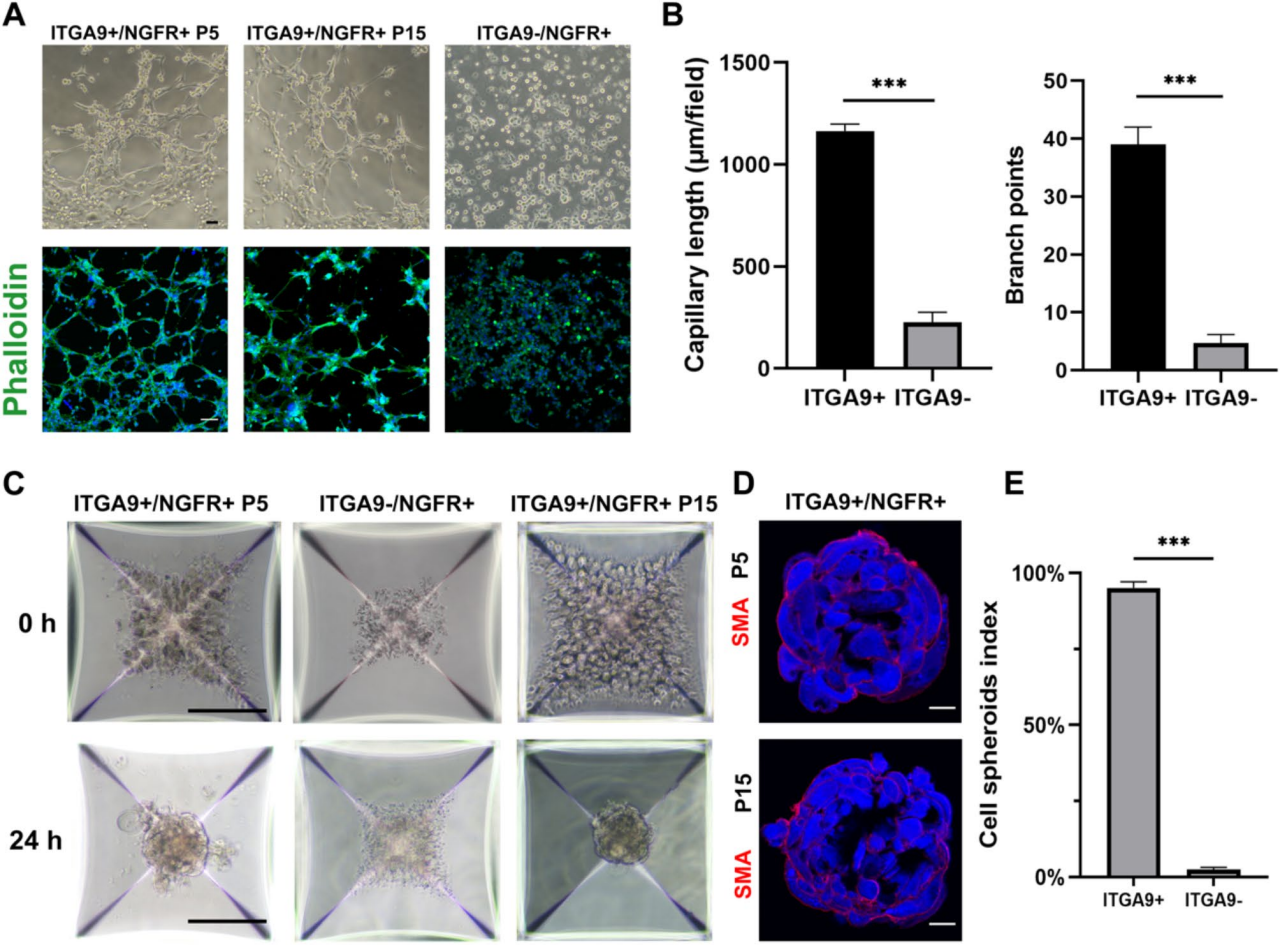
Characterisations of ITGA9+/NGFR + cells in vitro (**A**) Representative phase-contrast micrograph of cells shows the difference of tube-forming ability. Immunofluorescence staining of phalloidin show the cytoskeleton of ITGA9+/NGFR + and ITGA9-/NGFR + cells. (**B**) Comparison of the capillary length and branch points between ITGA9+/NGFR + and ITGA9-/NGFR + cells. ***P < 0.001. (**C**) ITGA9+/NGFR + and ITGA9-/NGFR + cells in 3D microwell culture system. (**D**) Immunofluorescence staining of PTM-specific marker SMA show significant difference of cell spheroids index between ITGA9+/NGFR + and ITGA9-/NGFR + cells (**E**). ***P < 0.001. The scale bar represents 20 μm

To further evaluate the purity of the cells, the expression of the markers of PTMs and ALCs were examined by PCR analysis at P5. The ITGA9+/NGFR + cells expressed PTM-specific genes (ACTA2, DCN and GDNF) (Fig. 3D) and qPCR showed that relative ACTA2 expression was not reduced after 15 passages of cells (Fig. S5A). It was also found that the concentration of PTM-specific secretory factors (PGE2, MCP-1, IL6) in the culture medium increased significantly compared with DF12 medium (Fig. 3E). Moreover, ITGA9+/NGFR + cells showed the tubular formation characteristic of blood vessel cells under the light microscope after phalloidin staining (Fig. 4A). We also found that the ITGA9+/NGFR + cells had the ability to aggregate and form spheroids in vitro (Fig. 4C&D). These two properties were still present when the cells reached passage 15 (Fig. 4A&C) and ITGA9-/NGFR + cells show completely different properties (Fig. 4B&E). ITGA9-/NGFR + cells did not show the tubular formation characteristic based on the evidences from light microscopy, fluorescence, capillary length and branch points (Fig. 4A&B), either the ability of cell aggregation or cell spheroids-forming in vitro (Fig. 4C&E).

### Long-time culture of ITGA9-/NGFR + cells in vitro

**Fig. 5:**
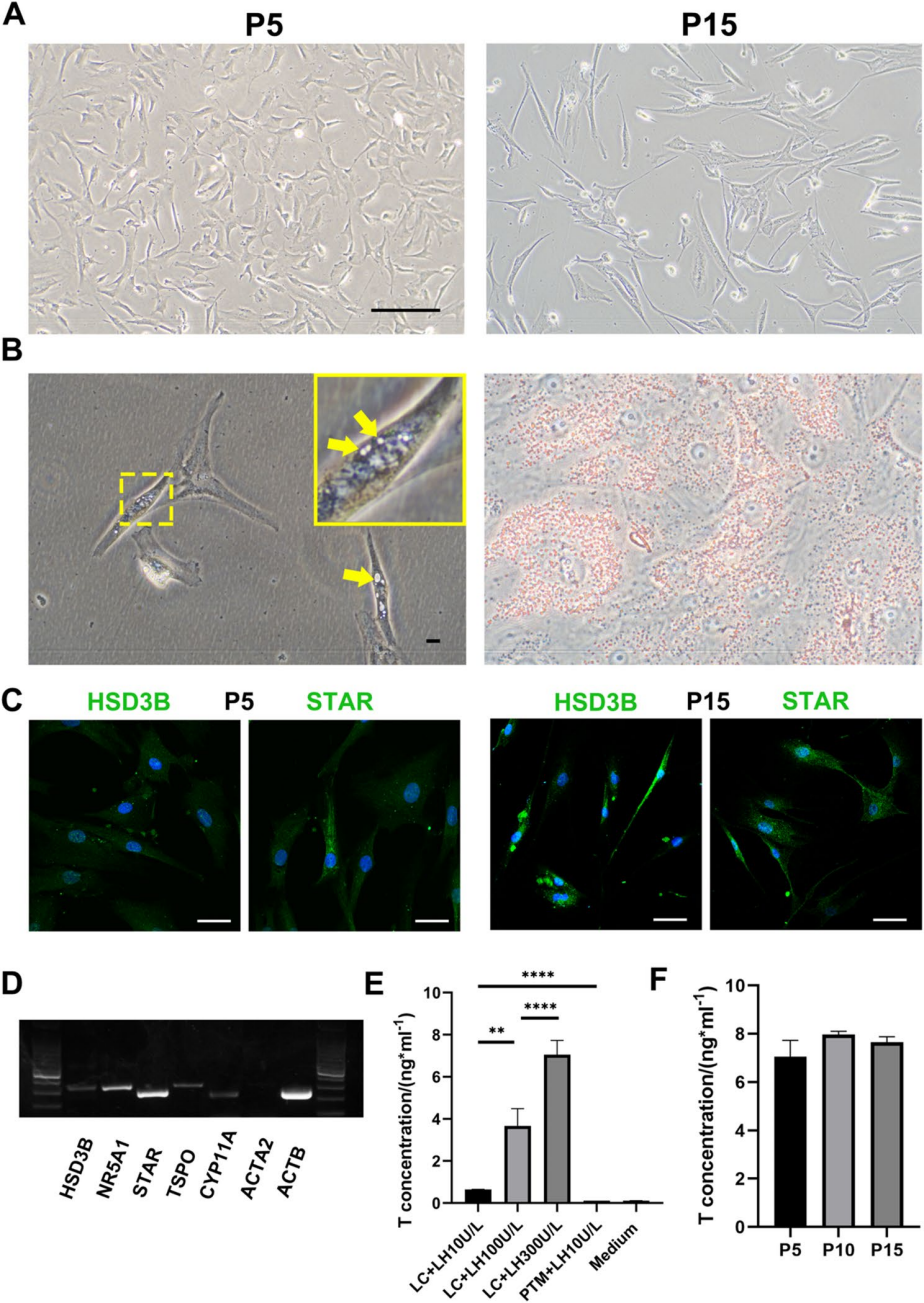
Identification of ITGA9-/NGFR + cells in vitro*.* (**A**) There was no obvious change in cell morphology after 15 passages under light microscope. (**B**) Intracellular lipid droplets can be found in ITGA9-/NGFR + cells by Oil Red O staining. (**C**) ITGA9-/NGFR + cells were positive for HSD3B, STAR. (**D**) RT-PCR show that ITGA9-/NGFR + cells express LC-specific genes of testosterone synthesis pathways (HSD3B, NR5A1, STAR, TSPO, CYP11A), but do not express PTM-specific genes. (**E**) By stimulating cells with different concentrations of LH, as the LH concentration in the medium increased, the level of testosterone secreted by ITGA9-/NGFR + cells increased correspondingly, while ITGA9+/NGFR + cells did not have this feature. (**F**) The testosterone secretion of LCs did not decrease significantly after 15 passages. The scale bar represents 20 μm

ITGA9-/NGFR + cells could be passaged to the 15th passage, and these cells grew in a fusiform (Fig. 5A). Compared with ITGA9+/NGFR + cells, the cell area, perimeter, and area-to-perimeter ratio of ITGA9-/NGFR + cells were significantly different, and the two types of cells exhibited completely different morphologies and cell refractive indices (Fig. S4). Moreover, intracellular lipid droplets could be seen in ITGA9-/NGFR + cells under a high magnification microscope by Oil Red O staining (Fig. 5B). ITGA9-/NGFR + cells were positive for HSD3B, STAR (Fig. 5C) and were negative for markers of PTMs and Sertoli cells (Fig. S2B). ITGA9-/NGFR + cells at passage 15th are still positive for STAR and HSD3B (Fig. 5C). At mRNA level, ALCs expressed key genes in testosterone synthesis pathways (HSD3B, NR5A1, STAR, TSPO, CYP11A) (Fig. 5D) and qPCR showed that relative CYP11A expression was not reduced after 15 passages of cells (Fig. S5B). ITGA9-/NGFR + cells were cultured with different concentrations of LH, as the LH concentration in the medium increased, the level of testosterone secreted by ITGA9-/NGFR + cells increased correspondingly, while ITGA9+/NGFR + cells did not show this feature (Fig. 5E). Moreover, the ITGA9-/NGFR + cells still were able to secrete testosterone in vitro at 10th and 15th passages, and the secretion capacity of the cells was not significantly different between the two different generations (Fig. 5F).

## Discussion

The somatic cells of the testis play an important role in spermatogenesis, hormone regulation and testicular immunity. The age-related decline in testosterone synthesis in the testicular LCs is a direct cause of hypogonadism in older men. Sperm transport is an established function of PTMs through their contractile-relaxing property, whereby they move sperm to the *rete testes* [24]. PTMs also secrete a number of factors through which they can influence testicular functions [25]. LCs and PTMs have the same origin and share many similarities. Although LCs can be separated from germ cells and Sertoli cells by differential adherence, flow cytometric sorting and/or gradient density centrifugation. However, separating the two cell types with high purity and recovery rate is challenging. In this study, we found that ITGA9 and NGFR can serve as specific surface markers for the identification and isolation of the two cell types in humans.

To date, only a few studies have reported on methods for isolating adult Leydig cells. The most promising method is gravity sedimentation together with the Percoll gradient. However, this method requires massive testicular tissue and the purity of the cells cannot be guaranteed [10, 26–28]. The generation of human adult LCs in vitro is usually induced by SLCs. However, the precursors of both LCs and PTMs lines express the same marker proteins, such as NGFR in developing animals [4]. The cellular localization of NGFR in human testis remains controversial, and there are many conflicting results about its expression. NGFR has been reported to be expressed in spermatocytes and Sertoli cells in rodents [29, 30] and as a surface marker for the identification and isolation of human SLCs [6, 31]. In some previous studies, NGFR was found to be present in germ cells in adult human testis [32–34]. Other studies also showed that the expression of NGFR is restricted to the vascular endothelial cells (VECs) [9]. Thus, germ cells, VECs or ALCs could be falsely sorted as SLCs if NGFR is used as a marker for the isolation of human SLCs, and induction of differentiation by SLCs will also cause cross-contamination of PTMs and LCs. The single cell transcriptome data of testicular tissues show that NGFR is mainly expressed by ALCs and PTMs. In our study, we allowed the cell suspension to adhere to the culture surface before the FACS step. This effectively removes non-adherent cells such as germ cells and effectively improves the purity of ALCs and PTMs. We found that these cells can be cultured for more than 15 passages.

Testosterone is produced by ALCs under LH regulation. LH induces a rapid synthesis of StAR which helps to transfer cholesterol crossing mitochondrial membranes to increase testosterone [35, 36]. In the current study, we found that the ITGA9-/NGFR + cells could secrete considerable amount of testosterone in response to LH in a concentration-dependent manner. These cells also express the LCs-specific genes and markers, and these expressions and cellular morphology differences are distinguished from PTMs [11]. We found that ITGA9-/NGFR+ cells expressed all steroidogenic pathway proteins, and also expressed the secreted marker of ALCs in vitro, which is consistent with the previously in vivo results [5]. At the same time, we found that when the cells were passaged to the 15th passage, testosterone secretion level of ALCs did not decrease significantly compared with the 5th passage, suggesting that the long-term culture of ALCs did not cause significant cell senescence. This is different from the decreased testosterone secretion of ALCs during aging process in vivo, which may be due to the different environment of cells both settings, or due to the decrease in the number of ALCs in vivo during aging.

Although PTMs were isolated from small testicular fragments of patients and studied in vitro [12, 13]. These methods would inevitably contaminate the PTMs with other somatic cells, such as SCs, LCs, VECs, as being shown by our repeating experiment of the previous protocols. The FACS technique in this study effectively improves the purity of PTMs isolated, which could benefit greatly to future PTMs researches. At the same time, we also found that it took a long time to generate enough peritubular cells for passage (over two weeks) through the previously reported method. However, it took only about 1 week by the current protocol and each generation of cells can reach about 90% fusion after 3–4 days of culture and can be passaged. The method also helped to validate many specific markers of PTMs such as SMA and GDNF. At the same time, this study also confirms some of the previous findings about PTM-specific secretion of inflammation-related factors, such as PGE2, MCP-1 and IL6 [11, 12, 14]. Expressions of these factors were all observed by the current study. However, PTMs senescence after long-term culture has been reported in previous studies in non-human primate and human [37, 38]. Our study did not find any obviously cellular senescence even after long-term culture (15 passages), which is different from previous researches.

Tubule formation assays are commonly used for studying the process of angiogenesis. During the assay, VECs differentiate, directionally migrate to align, branch, and form the tubular polygonal networks of blood vessels [39]. The critical step of forming new capillaries is critical to angiogenesis and the entire process is ultimately dependent on the morphogenesis of ECs into new blood vessels [40]. Our study compared the tubule-forming ability of the ALCs and PTMs. We found that PTMs showed the ability to form tubule-like structures similar to VECs, but such cells and vascular cells do not co-localize according to the immunofluorescence staining. Prior work based on juvenile human testes showed that LCs and PTMs share a common progenitor at pre-pubertal stage [3]. Particularly, several genes involved in tubule development are specifically expressed in these candidate progenitors, consistent with the initiation of tubule formation to create the testes cords at week 6 [41]. Our study shows that mature PTMs instead of LCs have characteristics of VECs when cultured in vitro. The reason could be that peritubular cells maintain more stem cell characteristics than Leydig cells in vitro. At the same time, VECs are also derived from mesoderm and their progenitor cells also have the ability of tubule formation [42], which means that PTMs cultured in vitro may have the characteristics of VECs and its progenitor cells in the 2D culture system.

Cell spheroid culture system is a valuable model for the study of progenitor cell behavior and physiology and the properties of PTMs in a 3D culture system have not been reported. We found that PTMs have the ability of forming cell aggregates in 3D culture system in vitro rather than in a 2D culture system, and this property can still be maintained after multiple passages, which differs from previous study [43]. This may be due to the co-interaction with Sertori cells in the 2D culture system. This property of PTMs can be used as natural scaffold for the testicular organoids which need microenvironment close to in vivo and spatial clues for cellular reorganization in vitro [44, 45]. Moreover, vascularization in 3D-tissue constructs is still a key challenge in the tissue engineering field since without proper vascularization necrosis constructs could happen inside thick 3D-tissue. Researchers have tried to control the capillary formation of endothelial cells in scaffolds [46] and reconstructed 3D-tissues [47, 48]. However, it is difficult to control the alignment of the reconstructed blood capillary. Our study found that peritubular cells exhibited different cellular properties in different culture systems, and at the same time possessed similar properties of vascular cells and could autonomously form 3D cell spheroids. It means PTMs might be used as a natural 3D culture scaffold, which can be further tested in future 3D culture research.

## Conclusions

ITGA9 and NGFR have been identified as specific markers of adult LCs and PTMs. ITGA9-/NGFR + cells isolated from human testes showed characteristics of ALCs with the ability of testosterone production, and ITGA9+/NGFR + cells showed characteristics of PTMs with the ability of tubular formation/cell aggregation in vitro. Both cells have the potential for long-term culture. Thus, the present study identifies promising discriminatory surface markers for both PTMs and adult LCs identification and isolation, which could benefit human PTM and ALC studies, such as cell characterization and organoid research in vitro, and promote potential clinical applications of the cells for managing male hypogonadism.

## Electronic supplementary material

Below is the link to the electronic supplementary material.

Supplementary Material 1

Supplementary Material 2

Supplementary Material 3

Supplementary Material 4

Supplementary Material 5

Supplementary Material 6

Supplementary Material 7

Supplementary Material 8

Supplementary Material 9

## Data Availability

No datasets were generated or analysed during the current study.
