# Supplementary figures and images for: Identification and isolation of human testicular peritubular myoid cells and Leydig cells by a combination of ITGA9 and NGFR
Source: Reprod Biol Endocrinol. 2025 May 31;23:82. doi: 10.1186/s12958-025-01389-w (PMC12125841; doi:10.1186/s12958-025-01389-w)

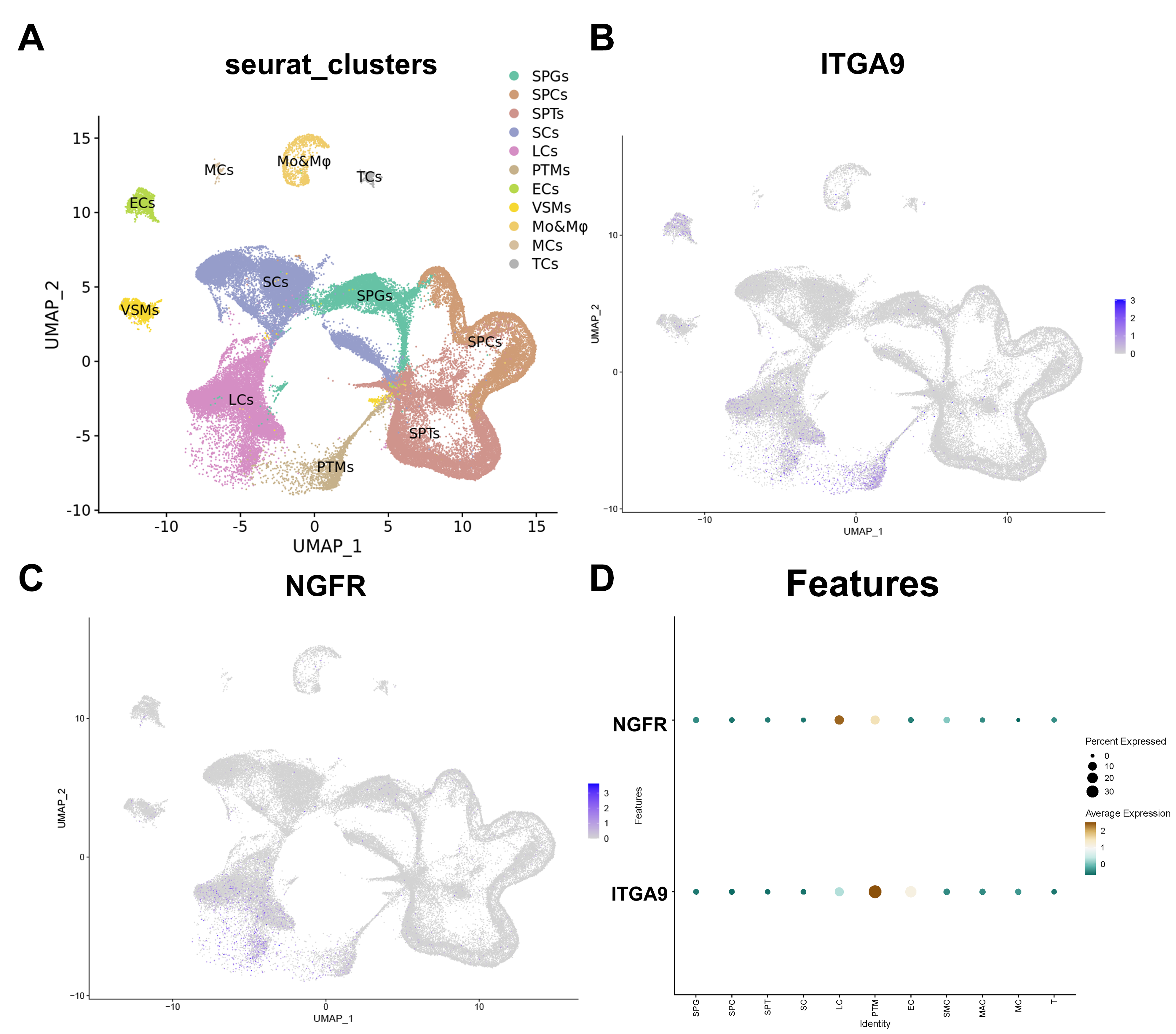

Supplement: Supplementary file 1 — Supplementary Material 1 [file 12958_2025_1389_MOESM1_ESM.tif]

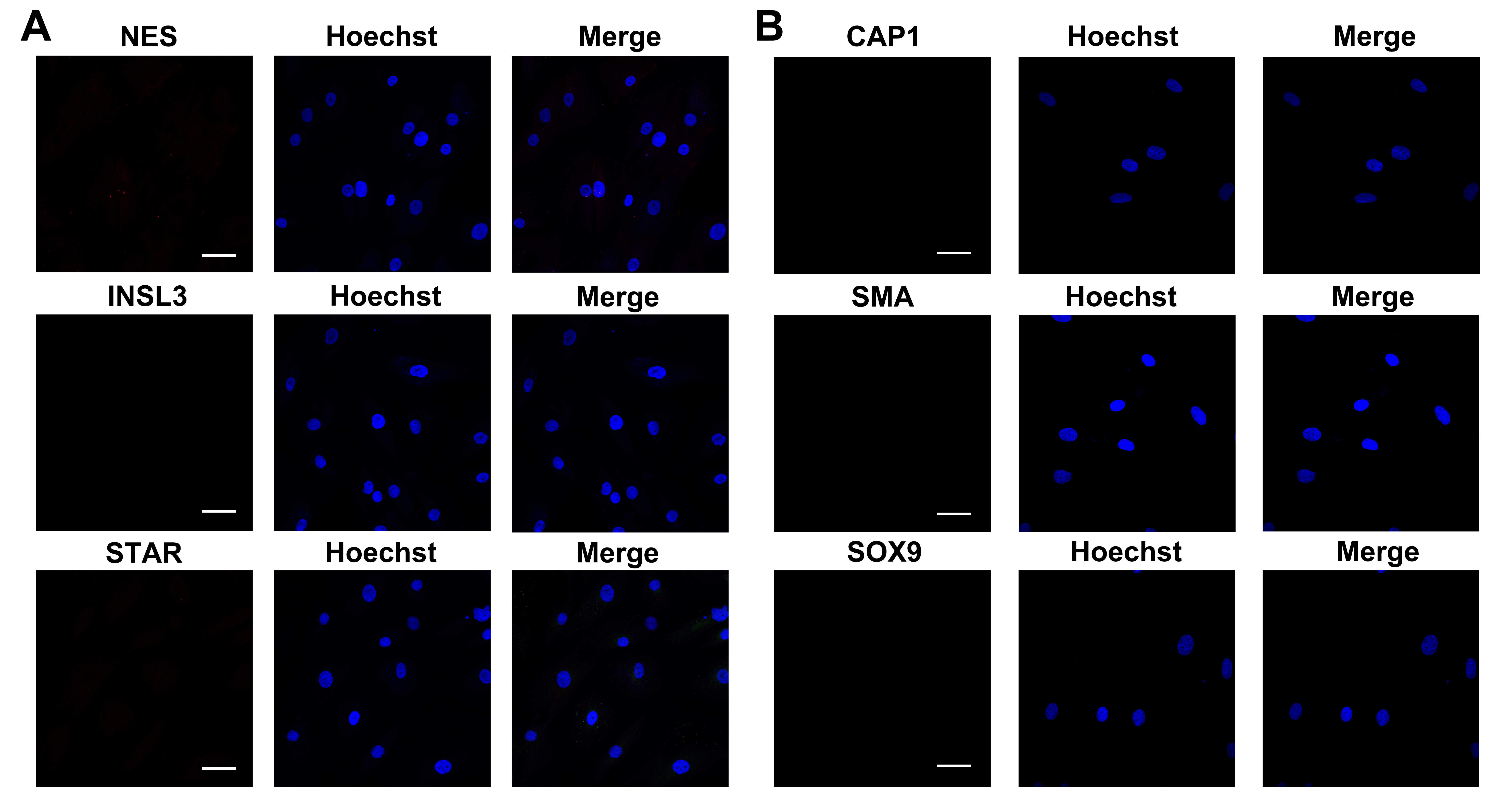

Supplement: Supplementary file 2 — Supplementary Material 2 [file 12958_2025_1389_MOESM2_ESM.tif]

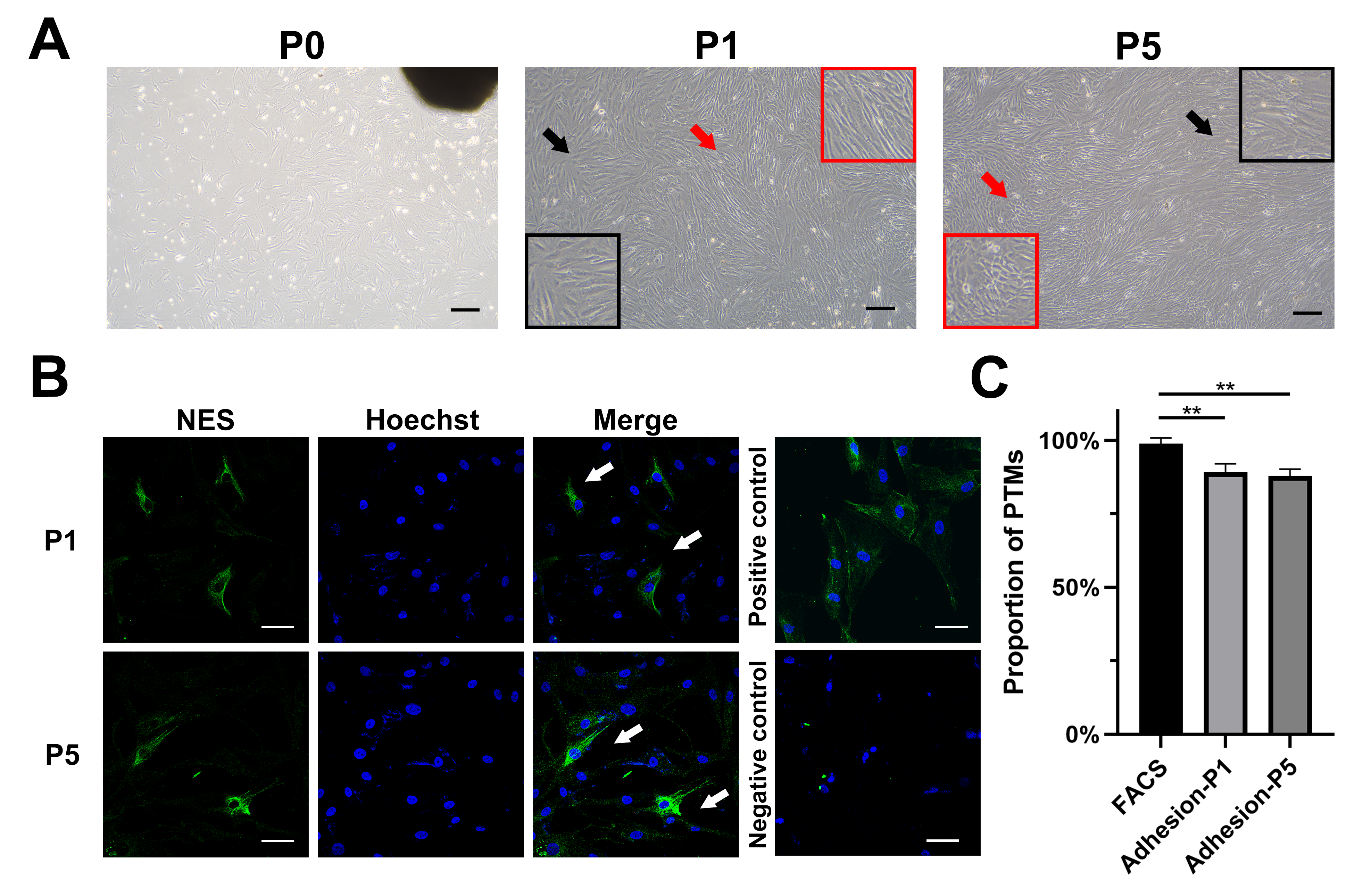

Supplement: Supplementary file 3 — Supplementary Material 3 [file 12958_2025_1389_MOESM3_ESM.tif]

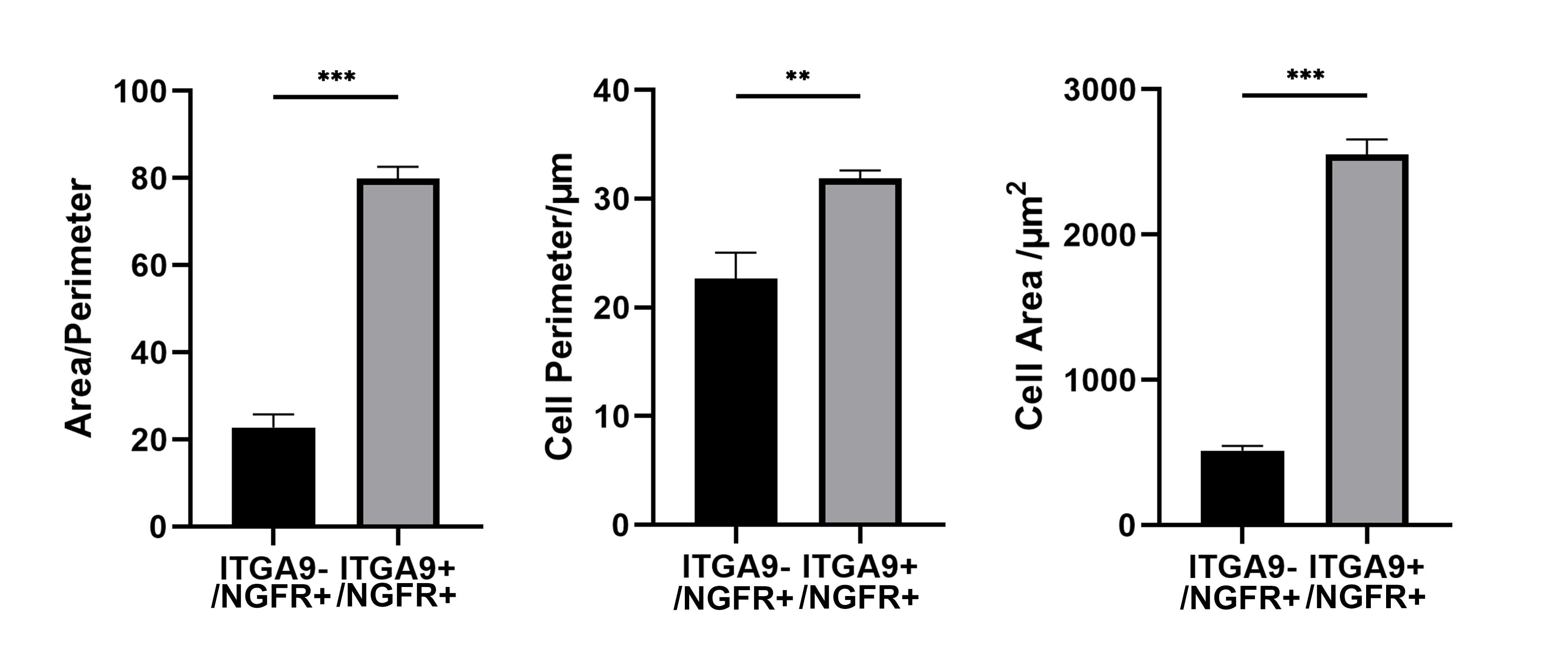

Supplement: Supplementary file 4 — Supplementary Material 4 [file 12958_2025_1389_MOESM4_ESM.tif]

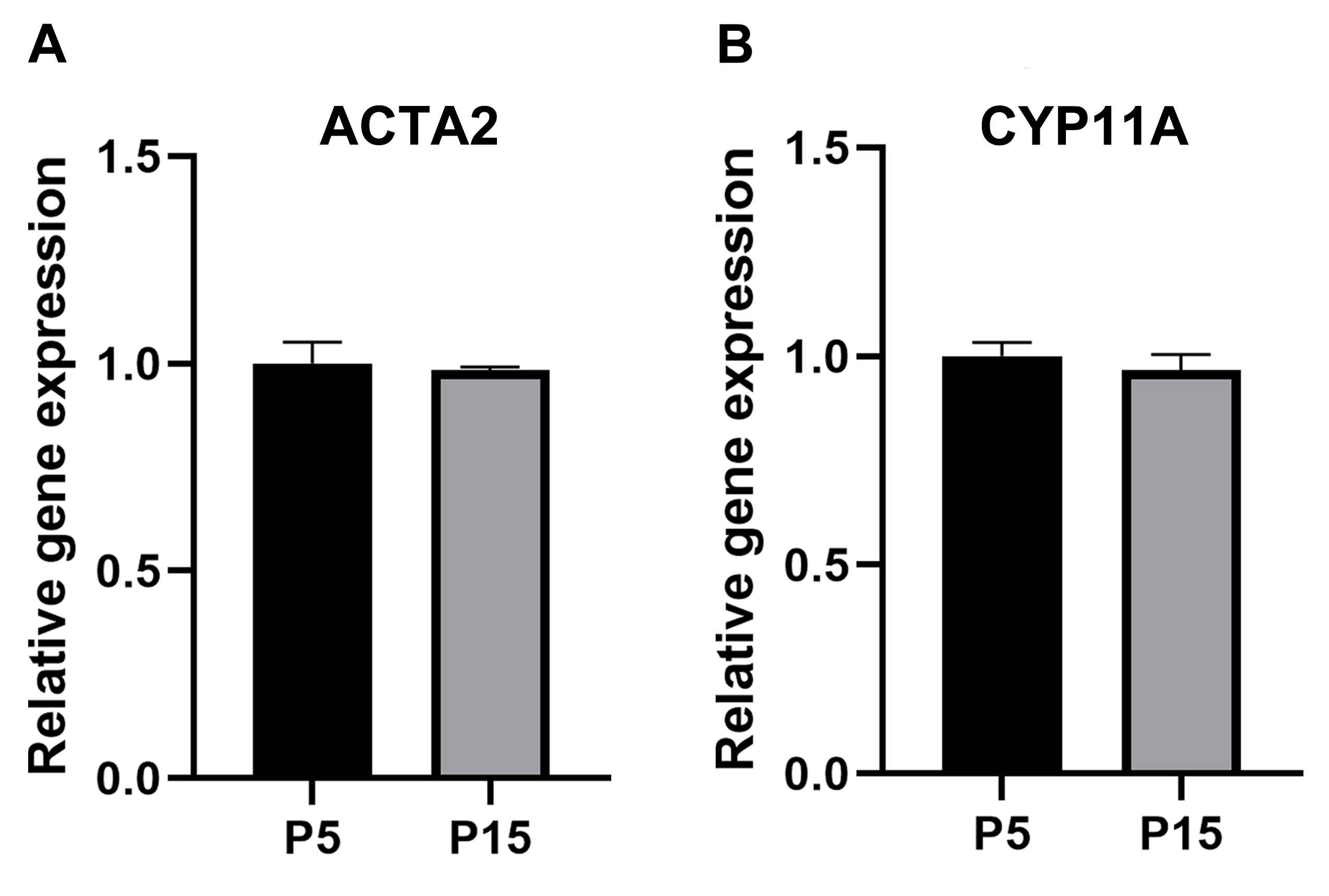

Supplement: Supplementary file 5 — Supplementary Material 5 [file 12958_2025_1389_MOESM5_ESM.tif]

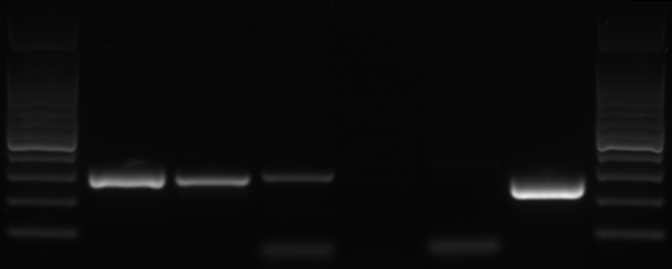

Supplement: Supplementary file 6 — Supplementary Material 6 [file 12958_2025_1389_MOESM6_ESM.tif]

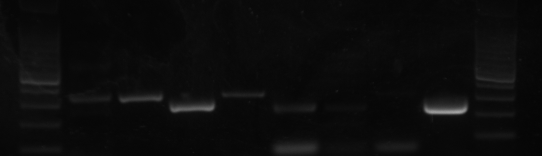

Supplement: Supplementary file 7 — Supplementary Material 7 [file 12958_2025_1389_MOESM7_ESM.tif]
