# Supplementary material for: Identification and isolation of human testicular peritubular myoid cells and Leydig cells by a combination of ITGA9 and NGFR
Source: Reprod Biol Endocrinol. 2025 May 31;23:82. doi: 10.1186/s12958-025-01389-w (PMC12125841; doi:10.1186/s12958-025-01389-w)
Supplement: Supplementary file 8 — Supplementary Material 8 [file 12958_2025_1389_MOESM8_ESM.docx]

Table S1. Primer sequences of genes used for RT-PCR.

| Gene | Forward primers (5'-3') | Reverse primers (5'-3') |
| --- | --- | --- |
| CYP11A | CCAGACCTTTCTGAGTGCCC | CTCCCTGTAAATCGGGCCAT |
| STAR | GGCTACTCAGCATCGACCTC | CTAAACACGAACCCCACCCA |
| TSPO | GCAGCCTAGACGGGTCTTAC | CCATGGCTGAGTAGAGCGTG |
| INSL3 | GCGACCGTGAGTTGCTACAG | TTTATGGTGCTGTGTGGCCT |
| HSD3B | AGGGAGCAATGAGTATGTGGC | ACAGTCAGCTTGGTCCTGTT |
| DCN | ATCTCAGCTTTGAGGGCTCC | AGATGGCATTGACAGCGGAA |
| GDNF | GTCACTGACTTGGGTCTGGG | TGGAGCCGGAGTCAGATACA |
| ACTA2 | GCCAAGCACTGTCAGGAATC | CCCAGTTGGTGATGATGCCA |
| ACTB | GATTCCTATGTGGGCGACGA | GCCACACGCAGCTCATTGTA |
| NR5A1 | TGGAGGGAAGGGGACAGATT | GCCACAGAGAGGGGATCAAC |
