# Supplementary material for: Identification and isolation of human testicular peritubular myoid cells and Leydig cells by a combination of ITGA9 and NGFR
Source: Reprod Biol Endocrinol. 2025 May 31;23:82. doi: 10.1186/s12958-025-01389-w (PMC12125841; doi:10.1186/s12958-025-01389-w)
Supplement: Supplementary file 9 — Supplementary Material 9 [file 12958_2025_1389_MOESM9_ESM.docx]

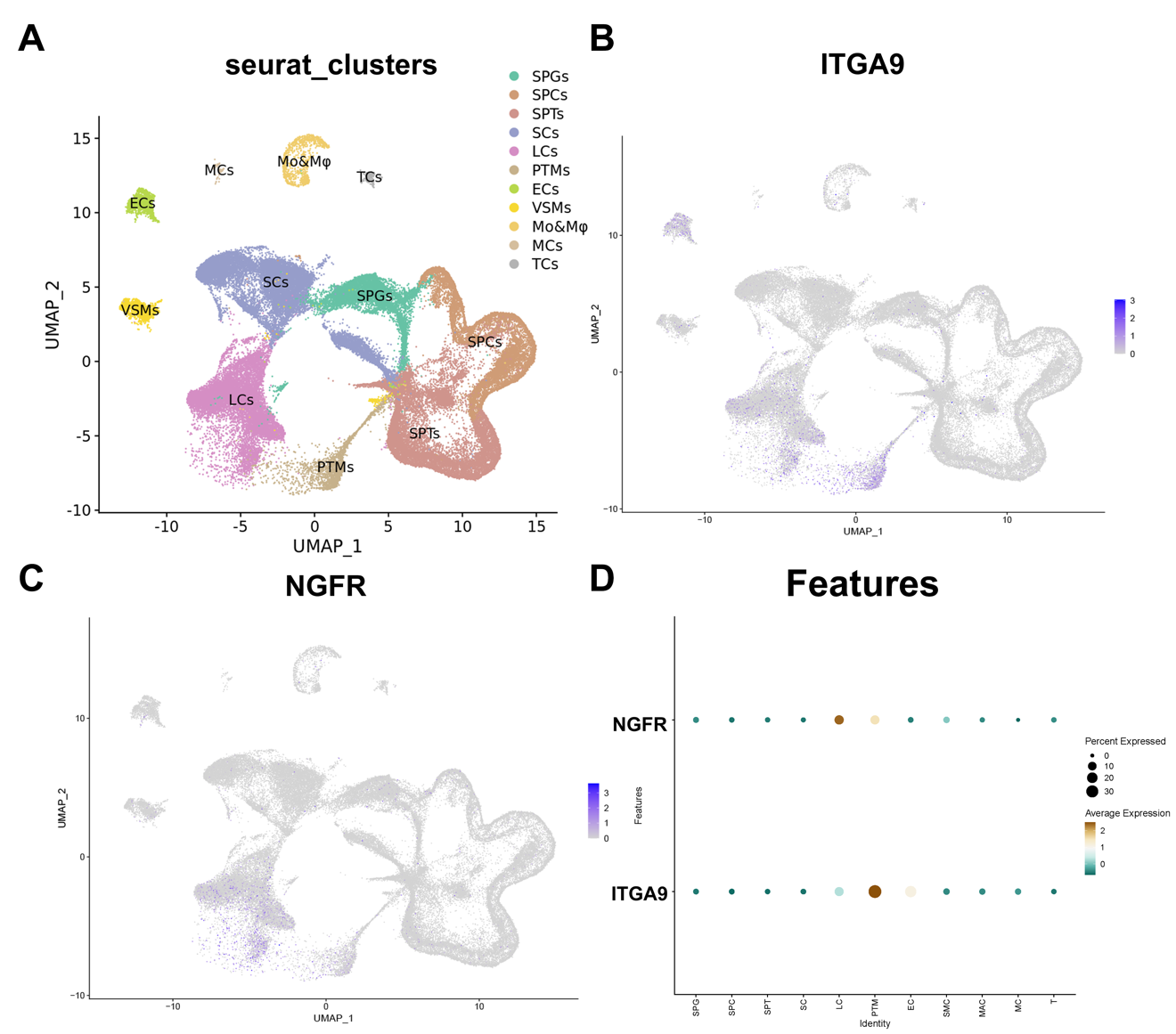


**Fig.S1** Expression profile of ITGA9 and NGFR in human adult testes. UMAP plots of all testicular cells (A) and ITGA9(B) /NGFR(C) expression. (D) Bubble matrix showing the ITGA9 and NGFR expression levels at each cell clusters. Cells are colored for types. UMAP, uniform manifold approximation and projection.


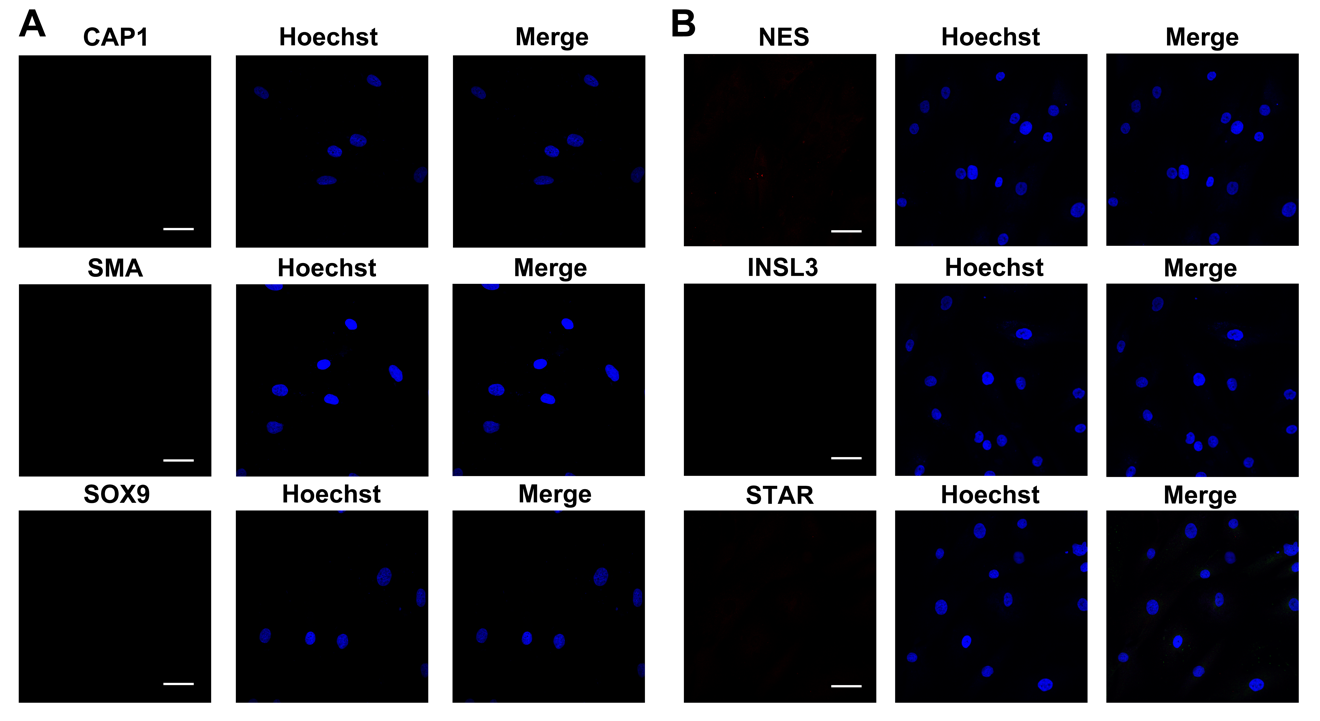


**Fig.S2** Negative results of immunofluorescence of ITGA9-/NGFR+ cells (A) and ITGA9+/NGFR+ (B). The scale bar represents 20 μm.

**
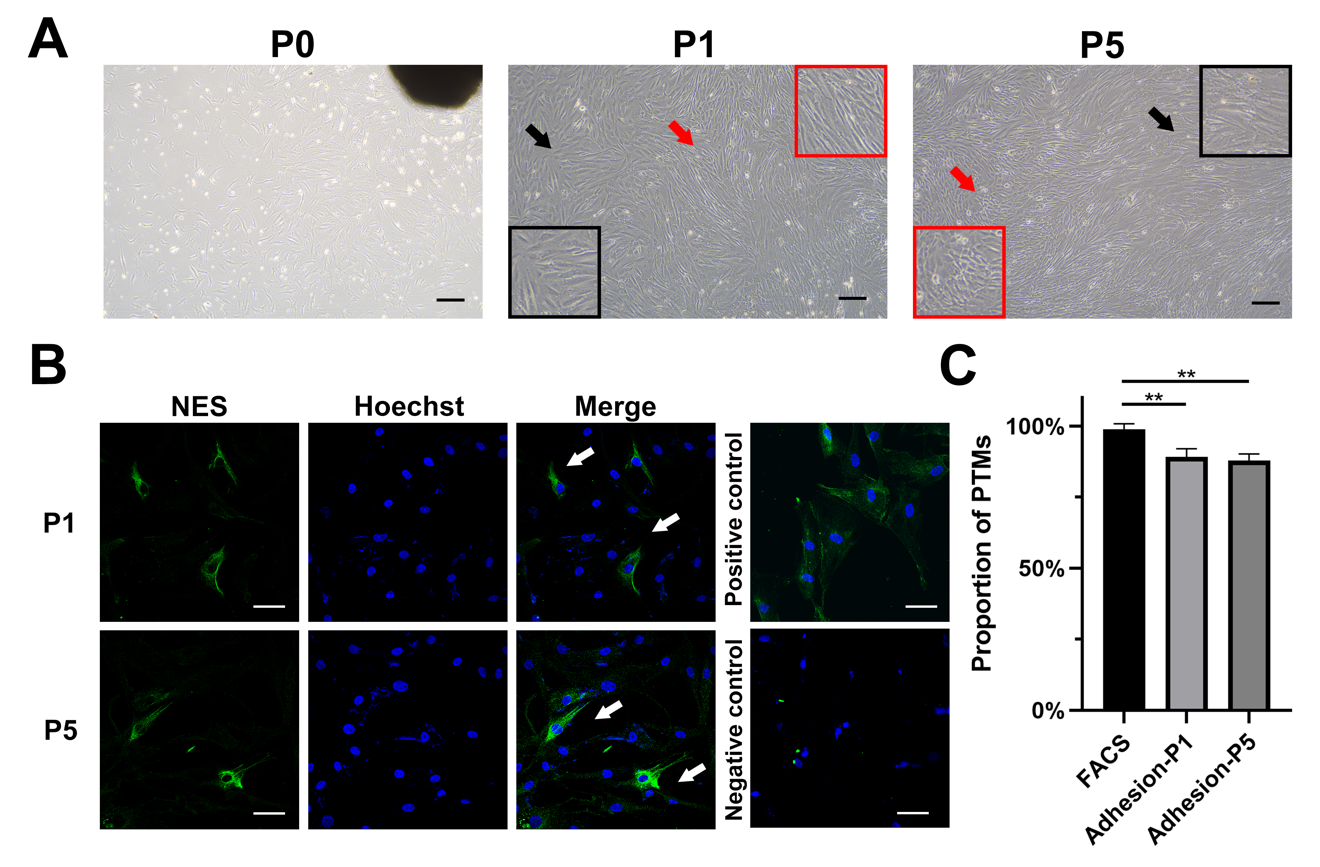
Fig.S3** Comparison of PTMs isolated through traditional tubule crawling method and FACS. (A) PTMs were isolated according to traditional tubule crawling methods. At least two different cell morphologies (indicated by red and black arrows) were seen in the 1st and 5th passages. (B) NES-positive cells were seen in the cells by immunofluorescence staining. (C) Comparison of the purity of peritubular cells isolated by the traditional method and FACS. **P<0.01. The scale bar represents 20 μm.


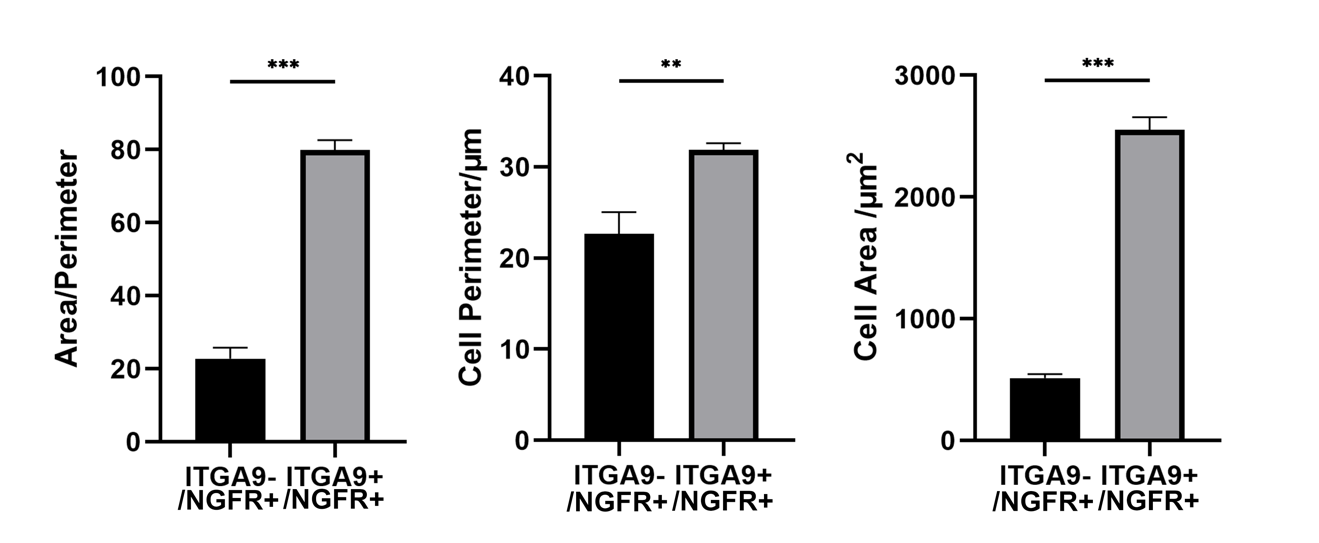


**Fig.S4** Comparison of perimeter, area and perimeter-area ratio of ITGA9+/NGFR+ and ITGA9-/NGFR+ cells.


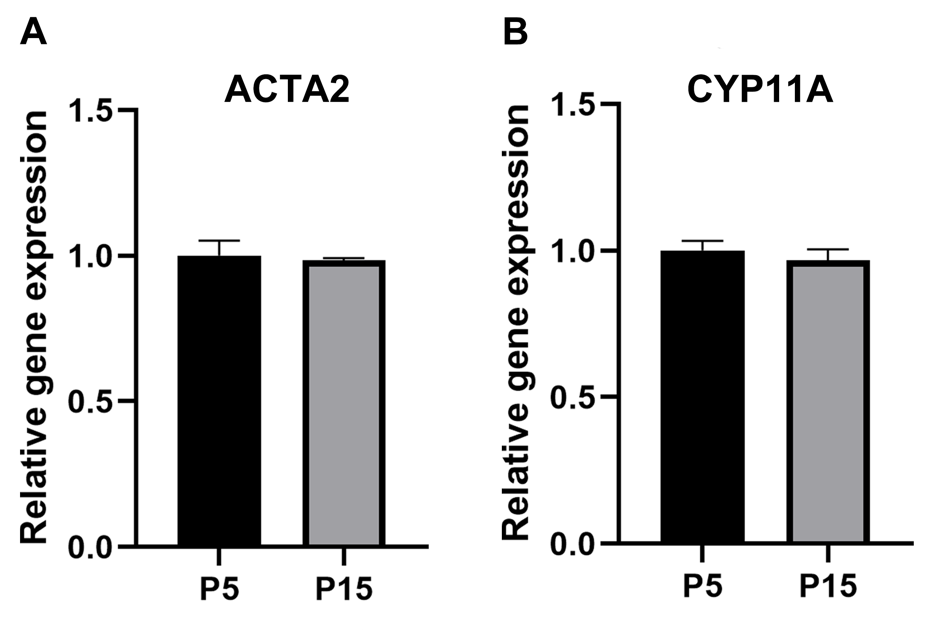


**Fig.S5** Comparison of mRNA expression of markers in P5 and P15 cells. (A) ITGA9+/NGFR+ cells. (B) ITGA9-/NGFR+ cells.
